# Between-Center Variation in Outcome After Endovascular Treatment of Acute Stroke: Analysis of Two Nationwide Registries

**DOI:** 10.1161/CIRCOUTCOMES.121.008180

**Published:** 2022-01-31

**Authors:** Paula M. Janssen, Katrine van Overhagen, Jan Vinklárek, Bob Roozenbeek, H. Bart van der Worp, Charles B. Majoie, Michal Bar, David Černík, Roman Herzig, Lubomir Jurák, Svatopluk Ostrý, Robert Mikulik, Hester F. Lingsma, Diederik W.J. Dippel

**Affiliations:** Department of Neurology (P.M.J, K.v.O., B.R., D.W.J.D.), Erasmus MC University Medical Center Rotterdam, the Netherlands.; Department of Radiology and Nuclear Medicine (B.R.), Erasmus MC University Medical Center Rotterdam, the Netherlands.; Department of Public Health (H.F.L.), Erasmus MC University Medical Center Rotterdam, the Netherlands.; International Clinical Research Center, Department of Neurology, St Anne’s University Hospital, Brno, Czech Republic (J.V., R.M.).; Faculty of Medicine at Masaryk University, Brno, Czech Republic (J.V., R.M.).; Brain Center, Department of Neurology and Neurosurgery, University Medical Center Utrecht, the Netherlands (H.B.v.d.W.).; Department of Radiology and Nuclear Medicine, Amsterdam University Medical Center, location AMC, the Netherlands (C.B.M.).; Department of Neurology, University Hospital Ostrava, Czech Republic (M.B.).; Faculty of Medicine at University Ostrava, Czech Republic (M.B.).; Masaryk Hospital Ústí nad Labem - KZ a.s., Comprehensive Stroke Center, Department of Neurology, Ústí nad Labem, Czech Republic (D.C.).; Comprehensive Stroke Center, University Hospital Hradec Králové, Czech Republic (R.H.).; Charles University Faculty of Medicine in Hradec Králové, Hradec Králové, Czech Republic (R.H.).; Regional Hospital Liberec, Neurocenter, Liberec, Czech Republic (L.J.).; Comprehensive Stroke Center, Department of Neurology, Hospital České Budějovice, a.s., České Budějovice, Czech Republic (S.O.).; Department of Neurosurgery and Neurooncology, First Faculty of Medicine, Charles University in Prague and Military University Hospital Prague (S.O.).

**Keywords:** hospitals, multicenter study, quality improvement, stroke, thrombectomy

## Abstract

Insight in differences in patient outcomes between endovascular thrombectomy (EVT) centers can help to improve stroke care. We assessed between-center variation in functional outcome of patients with acute ischemic stroke who were treated with EVT. We analyzed to what extent this variation may be explained by modifiable center characteristics.

**Methods:**

We used nationwide registry data of patients with stroke treated with EVT in the Netherlands and in the Czech Republic. Primary outcome was modified Rankin Scale score at 90 days as an indicator of disability. We used multilevel ordinal logistic regression to quantify the between-center variation in outcomes and the impact of patient and center characteristics. Between-center variation was expressed as the relative difference in odds of a more favorable modified Rankin Scale score between a relatively better performing center (75th percentile) and a relatively worse performing center (25th percentile).

**Results:**

We included a total of 4518 patients treated in 33 centers. Adjusted for patient characteristics, the odds of a more favorable outcome in a center at the 75th percentile of the outcome distribution were 1.46 times higher (95% CI, 1.31–1.70) than the odds in a center at the 25th percentile. Adjustment for center characteristics, including the median time between stroke onset and reperfusion per center, decreased this relative difference in odds to 1.30 (95% CI, 1.18–1.50, *P*=0.01). This translates into an absolute difference in likelihood of good functional outcome of 8% after adjustment for patient characteristics and to 5% after further adjustment for modifiable center characteristics.

**Conclusions:**

The considerable between-center variation in patient outcomes after EVT for acute ischemic stroke could be largely explained by center-specific characteristics, such as time to reperfusion. Improvement of these parameters may likely result in a decrease in center-specific differences, and an overall improvement in outcome of patients with acute ischemic stroke.

## Abstract 2

Supplemental Digital Content is available in the text.

**What Is Known**

- Variation in functional outcome of patients with acute ischemic stroke is only partially explained by patient characteristics, such as stroke severity and medical history, which are mostly unmodifiable.
- Modifiable characteristics of centers providing endovascular thrombectomy for acute ischemic stroke, for example number of endovascular thrombectomies performed or time to treatment, can differ between centers.

**What the Study Adds**

- In this international cohort of acute ischemic stroke patients treated with endovascular thrombectomy, functional outcome of patients differed substantially between centers and could be largely explained by center characteristics, such as time to reperfusion.
- Decreasing between-center variation in outcome by improving center-specific parameters may likely result in an overall improvement in outcome of patients with acute ischemic stroke.

Endovascular thrombectomy (EVT) improves outcomes in patients with acute ischemic stroke due to a large vessel occlusion in the anterior circulation.^1^ However, the implementation of EVT in clinical practice has been challenging because of changes required in local and regional workflows concerning acute ischemic stroke treatment.

Studies from the pre-EVT era have reported that outcomes of patients with acute ischemic stroke vary substantially between centers and that this variation can be partially explained by patient characteristics, but possibly also by specific center characteristics.^2–5^ However, the exact impact of center characteristics, such as center type and stroke volume, on between-center differences in outcome has remained uncertain. Insight in the potential causes of variation in outcomes between centers could contribute to the improvement of stroke care.

Little is known about the between-center variation in outcomes in patients treated with EVT. Variation in outcomes after EVT between centers can be caused by differences in patient population (eg, differences in stroke severity), but also by structural differences (eg, numbers of EVTs performed), or differences in processes (eg, times to treatment). Center-specific structural and process factors are largely modifiable contrary to patient characteristics. Insight into modifiable factors that could explain between-center variation in outcomes may inform improvements of EVT work processes and thereby improve patient outcomes. In this study, we aim to assess the between-center differences in functional outcomes of patients with acute ischemic stroke treated with EVT and to analyze to what extent variation in outcomes can be explained by modifiable center characteristics.

## Methods

### Study Population

We used data from 2 nationwide registries, one from the Netherlands (MR CLEAN [Multicenter Randomized Controlled Trial of Endovascular Treatment for Acute Ischemic Stroke in the Netherlands] registry), and the other from the Czech Republic (SITS-TBY [Safe Implementation of Treatments in Stroke-Thrombectomy] registry). Data from the MR CLEAN registry cannot be made available for purposes of reproducing the results or replicating the procedure, as no patient approval has been obtained for sharing coded data. Syntax and output files of statistical analyses will be made available upon reasonable request.

The MR CLEAN registry is a multicenter, prospective observational cohort study, which started directly after the last inclusion in the MR CLEAN trial,^6^ and comprises all consecutive acute ischemic stroke patients undergoing EVT in the Netherlands. Enrollment started with 16 centers that also participated in the MR CLEAN trial. Three centers started performing EVT later and added patients to the MR CLEAN registry. The MR CLEAN registry has previously been described in more detail.^7^ For the present study, we used data from patients who received EVT between March 2014 and November 2017.

SITS is an international collaboration founded by the Karolinska Institute and was set up as an instrument to enhance safe implementation of intravenous thrombolysis in acute ischemic stroke in clinical practice. The SITS-TBY registry contains prospectively collected data from acute ischemic stroke patients who were treated with EVT. This global registry consists of a standard data entry protocol and can be used by individual stroke centers to document thrombectomy data and to compare outcomes with other centers and countries. For the present study, we used SITS-TBY Registry data from patients treated between January 2014 and December 2017 in 15 centers in the Czech Republic.^8^

From both registries, we selected stroke patients aged 18 years or older with intracranial large vessel occlusion in the anterior circulation, who were treated with EVT within 6.5 hours after onset of symptoms or last seen well. Patients were excluded for the present study when information on one or more of these inclusion criteria was missing.

A central medical ethics committee evaluated the study protocol of the MR CLEAN registry and granted permission to perform the study as a registry, as the study required no additional interventions or procedures beyond those performed as usual care. Ethics approval was obtained from the local institutional review board for the analysis of the Czech data from the SITS-TBY registry. Study results are reported in accordance with the Strengthening the Reporting of Observational Studies in Epidemiology statement.^9^

### Data Collection

We collected baseline patient data on age, sex, previous ischemic stroke, diabetes, atrial fibrillation, hypertension, hypercholesterolemia, smoking status, prestroke modified Rankin Scale (mRS) score, baseline National Institutes of Health Stroke Scale (NIHSS) score, occlusion side, and occlusion segment on computed tomography angiography. The mRS is a 7 point disability scale ranging from 0 (no symptoms) to 6 (death). The NIHSS, ranging from 0 to 42, is a scale that quantifies stroke symptoms. A higher score indicates more and worse neurological deficits.

Data on workflow were intravenous alteplase treatment, transfer status (first presentation at a primary stroke center or at an intervention center), times from stroke onset to door of the first hospital, groin puncture, and reperfusion, time from door of the first hospital to groin puncture, and time from groin puncture to reperfusion. Onset of stroke was defined as the time of symptom onset or the time of last seen well. The definition of time of reperfusion or end of procedure slightly differed between the 2 registries. In the MR CLEAN registry, this was defined as the time of successful reperfusion, last contrast bolus, or end of procedure, while in the SITS-TBY registry, this was defined as the time of closure of the puncture site. The time of arrival at the door of the first hospital was collected for each patient. This means that for nontransferred patients, this time point refers to the arrival at the intervention center, and for transferred patients, this time point refers to the arrival at the primary stroke center.

Additional center characteristics were collected, including center type (university hospital, nonuniversity teaching hospital, or nonuniversity nonteaching hospital) and availability of endovascular treatment for intracranial aneurysms. In addition, for each center, we calculated the mean number of patients treated with EVT per 3 months using all registered patients in the study period without applying any of our inclusion criteria. Furthermore, we calculated the percentage of patients with an onset-to-groin puncture time of >390 minutes for each center, using only data of patients with the occlusion in the anterior circulation, as an indicator of guideline adherence.

The primary outcome was mRS score at 90 days, which we analyzed both ordinal as dichotomized. Other outcome data were NIHSS scores at 24 to 48 hours.

### Statistical Analysis

Because of the nested structure of the data, we used multilevel mixed-effects ordinal logistic regression with a random intercept for center to quantify the between-center variation in mRS scores at 90 days. To analyze the impact of patient characteristics and modifiable center characteristics on between-center variation in functional outcomes, the analysis was performed in a stepwise manner. We started with a model with adjustment for 12 patient characteristics: age, sex, previous ischemic stroke, diabetes, atrial fibrillation, hypercholesterolemia, hypertension, smoking, occlusion side, occlusion segment, baseline NIHSS score, and prestroke mRS score. Percentage of intravenous alteplase treatment and percentage of transferred patients were included as center characteristics (in the next model) and not as patient-level variables. Subsequently, center characteristics were added in 3 sets as fixed effects to the model. The first set of center characteristics concerned factors that are slightly modifiable: mean number of treated patients per 3 months, type of center, availability of endovascular treatment for intracranial aneurysms, percentage of patients treated with onset-to-groin puncture time >390 minutes, percentage of transferred patients, and percentage of patients receiving intravenous alteplase treatment. Then a second set of center characteristics, all concerning the times to treatment mentioned above, was added as fixed effect to the model. These characteristics are modifiable to a larger extent than those in the first set. Finally, the country variable was added as center characteristic to the model.

To help interpreting the estimated between-center differences in outcomes, we used the random intercept variance (tau2) to calculate the relative difference in odds of a more favorable outcome (ordinal mRS score) at 90 days between a center located at the 25th percentile of the outcome distribution (relatively worse performing center) and a center at the 75th percentile of the outcome distribution (relatively better performing center).^10^ This relative difference in odds was calculated with the formula: exp (2×0.67×tau). The value 2×0.67 is the Z value corresponding to the width of the 50% CI in a normal (Gaussian) distribution. The differences in between-center variation between the 4 steps of the analysis were each compared against the previous step with a likelihood ratio test.

The effect of each center characteristic on the between-center variation in outcomes, independently of the other center characteristics, was estimated by comparing a model with patient characteristics and all center characteristics against the same model with one specific center characteristic taken out with a likelihood ratio test. Common odds ratios were estimated for each center characteristic using a model including patient characteristics and all center characteristics.

Furthermore, we estimated the impact of the between-center variation in outcome for an average patient. For this estimation, we dichotomized mRS scores in 0–2 (good functional outcome) and 3–6 (poor functional outcome). An average patient was constructed by using the mean values for each patient characteristic, the mean values of the center characteristics availability of endovascular treatment for intracranial aneurysms and type of center, and the median values of the remaining center characteristics. For this average patient, the expected outcome at 90 days was calculated based on the model with adjustment for patient characteristics and again with adjustment for patient characteristics and all center characteristics.

To make unbiased estimates of regression effects, we substituted missing data using multiple imputation with 10 iterations, assuming missingness at random.^11,12^ The center variables percentage of transferred patients, percentage of patients treated with intravenous alteplase, median time from onset to door intervention center, median time from first door to groin puncture, and median time from groin puncture to reperfusion were computed from imputed variables. All reported baseline data are not imputed. We used Stata/SE statistical package version 15.1 (StataCorp, College Station, TX) for all analyses.

## Results

### Baseline Characteristics

**Figure 1.**
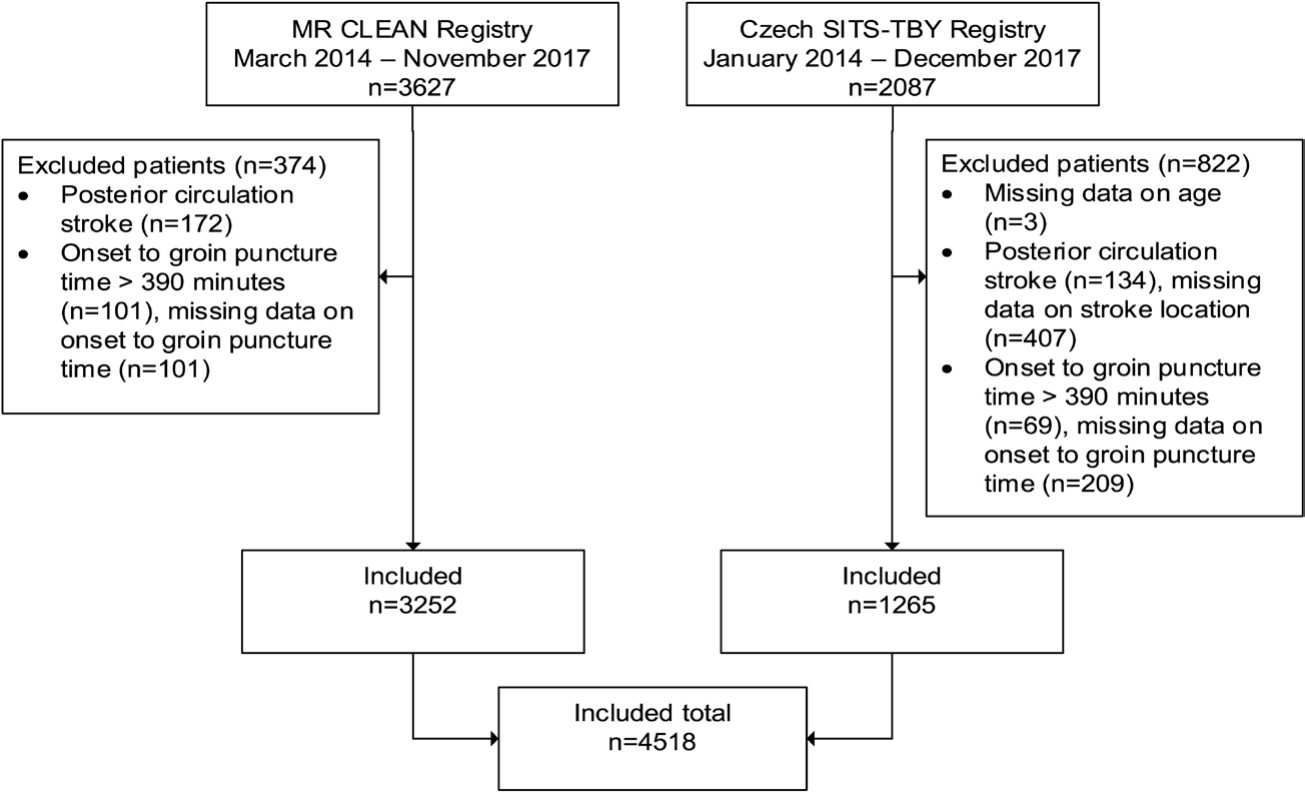
**Inclusion flowchart study population.** MR CLEAN indicates Multicenter Randomized Controlled Trial of Endovascular Treatment for Acute Ischemic Stroke in the Netherlands; and SITS-TBY, Safe Implementation of Treatments in Stroke-Thrombectomy.

A total of 4518 patients, from 19 centers in the Netherlands and 14 centers in the Czech Republic, were included (Figure 1). One center that reported 5 patients with incomplete data was excluded from the analysis.

The median age of the patients was 72 years (interquartile range [IQR] 62–80) and 2306 (51%) were male (Table 1). The prestroke mRS score was 0 or 1 in 3633 (82%) patients. The median NIHSS score at baseline was 16 (IQR, 11–19). The median time from onset to reperfusion was 243 minutes (IQR 195–306). The number of included patients receiving EVT per center in the study period ranged from 23 to 397 in the MR CLEAN registry, and from 1 to 195 in the Czech SITS-TBY registry. Median number of included patients per center for both registries together was 109 (IQR 58–192). The percentage of transferred patients per center ranged from 0 to 77% (median 62, IQR 45–68) in the MR CLEAN registry, and from 0% to 42% (median 16, IQR 4–33) in the Czech SITS-TBY registry (Table 2).

**Table 1.**
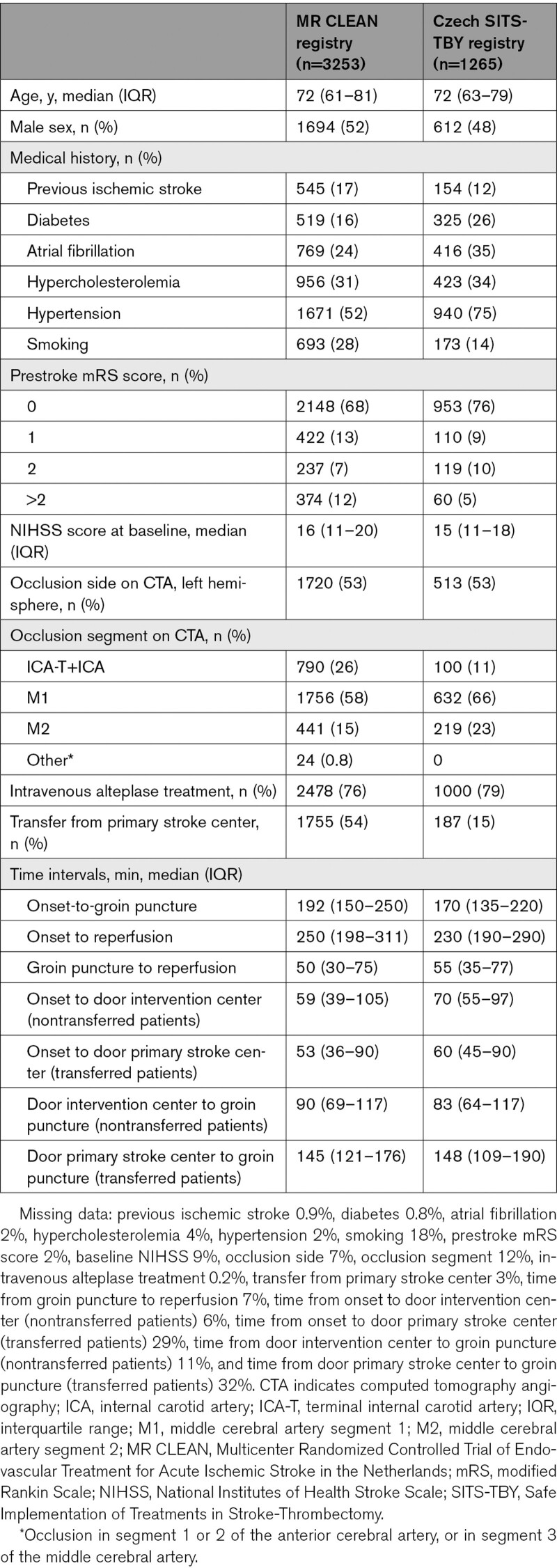
Baseline Patient Characteristics

**Table 2.**
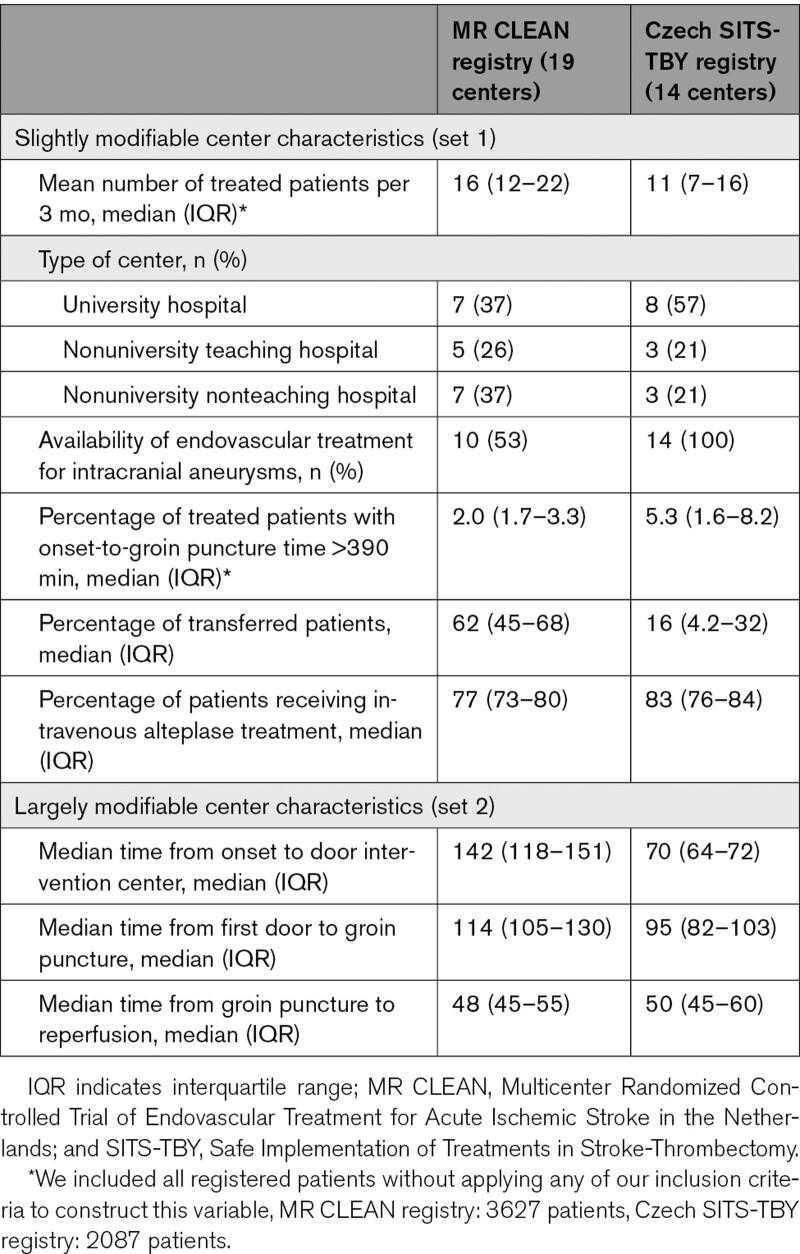
Baseline Center Characteristics

At 90 days, 1561 (42%) of 3696 patients had a good functional outcome (mRS score 0–2). The median NIHSS score at 24 to 48 hours was 9 (IQR, 3–16). Data on the mRS score at 90 days were missing in 822 (18%) patients and data on the NIHSS score at 24 to 48 hours in 753 (17%) patients. Information on both outcome parameters was missing in 210 (4.6%) patients.

### Between-Center Variation in Outcome

**Figure 2.**
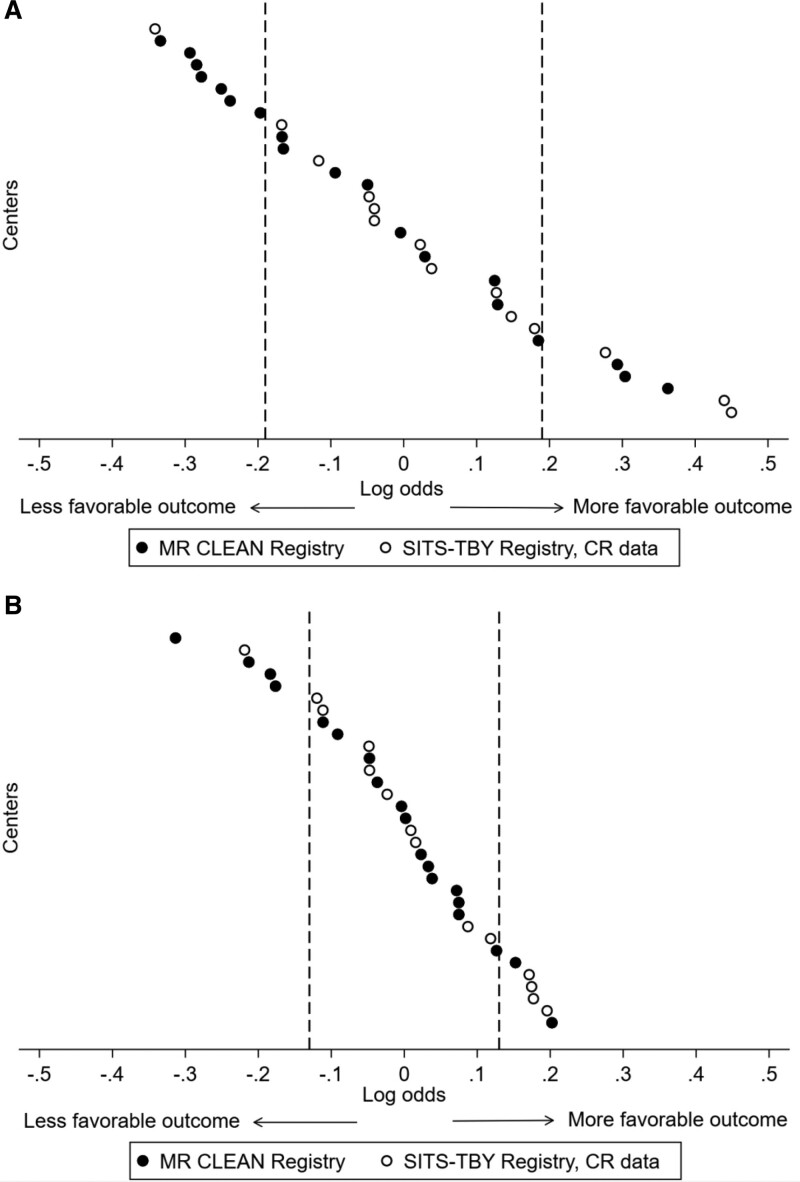
**Effect of center characteristics on between-center variation in outcomes after endovascular treatment for acute ischemic stroke.** Log odds of outcome, measured with the modified Rankin Scale at 90 d, calculated for each center and compared with the average, with adjustment for patient characteristics (**A**), and again with adjustment for patient and center characteristics (**B**). Each dot represents one center and centers are ranked from relatively worse to better performance. The dashed lines represent the 25th and 75th percentile of the outcome distribution. Log odds of −0.19 and 0.19 refer to odds ratios of 0.83 and 1.21, respectively (**A**), and log odds of −0.13 and 0.13 refer to odds ratios of 0.88 and 1.14, respectively (**B**). CR indicates Czech Republic; MR CLEAN, Multicenter Randomized Controlled Trial of Endovascular Treatment for Acute Ischemic Stroke in the Netherlands; and SITS-TBY, Safe Implementation of Treatments in Stroke-Thrombectomy.

Regarding the between-center comparisons, the odds of a more favorable mRS score at 90 days were 1.46 times higher (95% CI, 1.31–1.70) in a center at the higher end (75th percentile) of the outcome distribution than in a center at the lower end (25th percentile), after adjustment for patient characteristics (Table 3). After addition of the first and second set of center characteristics to the model, which included logistics parameters, this relative difference in odds decreased to 1.30 (95% CI, 1.18–1.50; *P*=0.01; Figure 2). When we also adjusted for country, this relative difference in odds between centers at the 25th and 75th percentile of the outcome distribution was 1.26 (95% CI, 1.15–1.47; *P*=0.10). To adjust for time-dependent changes in patient management not captured in our primary analysis, we added a time factor (year of intervention) to the primary multivariable analysis. Results were similar, and can be found in Table S1.

**Table 3.**
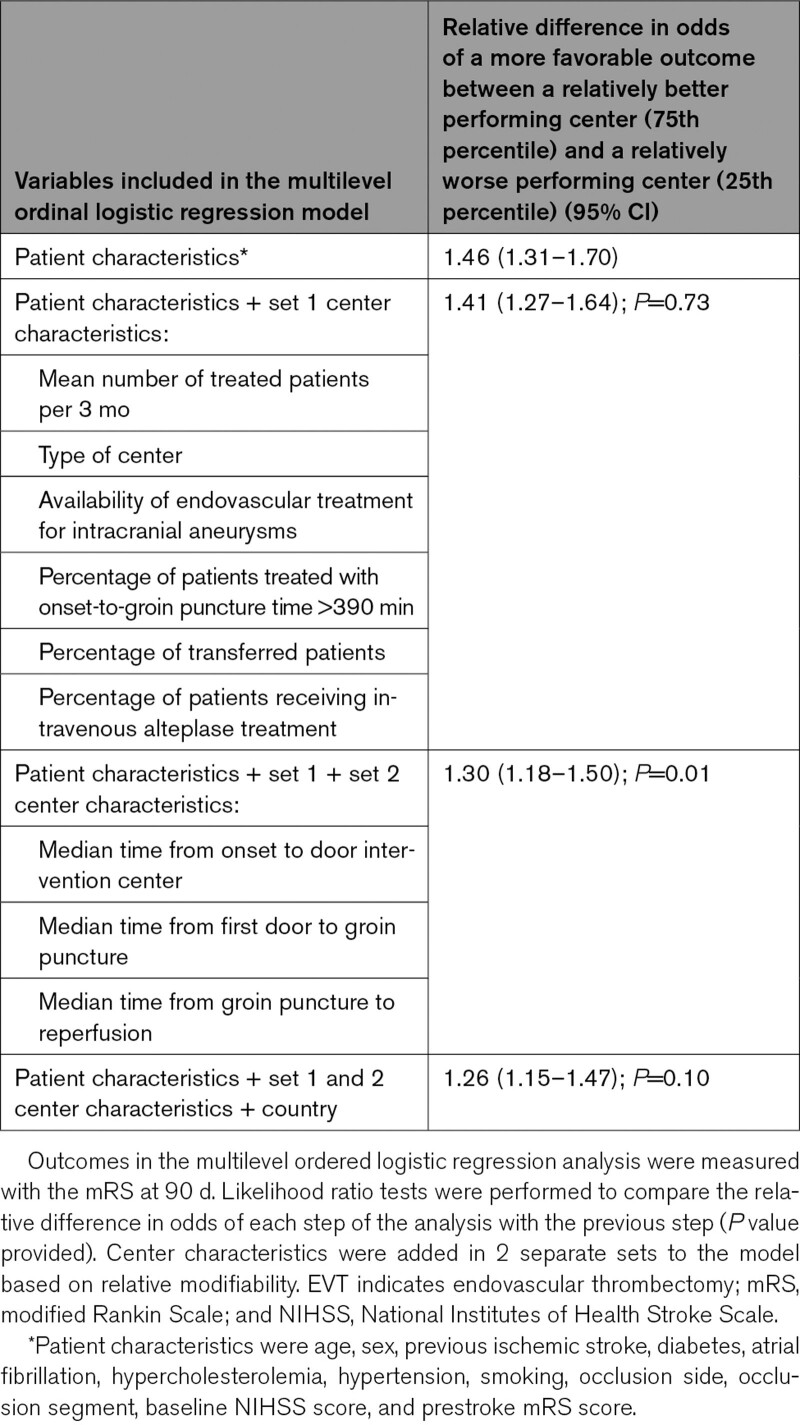
Effect of Combined Center Characteristics on Between-Center Variation in Outcome After EVT for Acute Ischemic Stroke

Of the center characteristics, only the median time from groin puncture to reperfusion had a statistically significant effect on the between-center variation in outcome, independently of the other center characteristics (Table 4). For every 10 minutes increase of the median time from groin puncture to reperfusion of an intervention center, the odds on a more favorable score on the mRS decreased with 12% (odds ratio, 0.88 [95% CI, 0.80–0.98]).

**Table 4.**
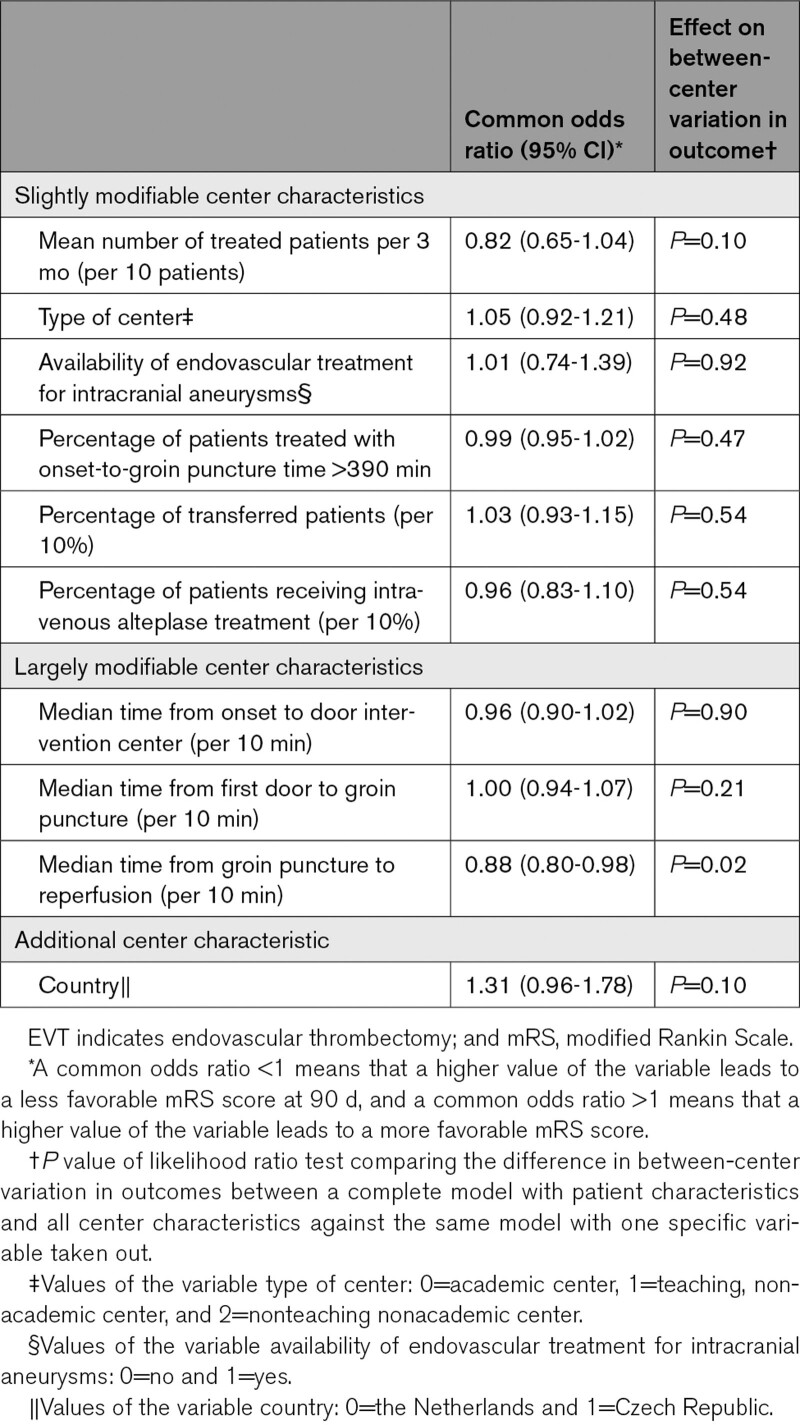
Effect of Each Center Characteristic on Between-Center Variation in Outcome After EVT for Acute Ischemic Stroke

**Figure 3.**
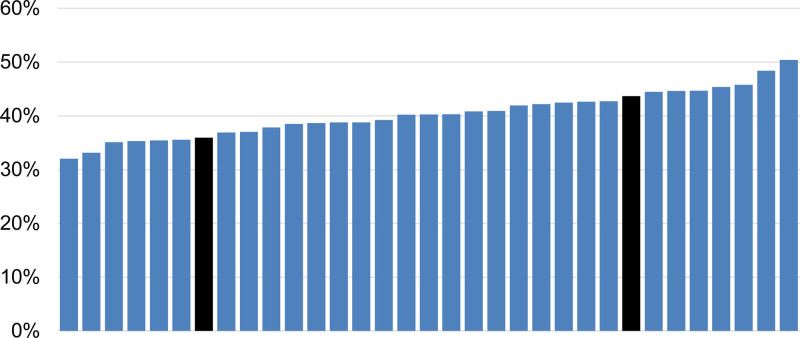
**Estimated frequency of good functional outcome (modified Rankin Scale [mRS] score 0–2) for an average patient for each center.** Each bar represents one center. The black bars represent the centers at the 25th and 75th percentile of the outcome distribution, corresponding to an interquartile range of the relative frequencies to achieve good functional outcome of 36%–44%, adjusted for patient characteristics.

The estimated frequency of good functional outcome (mRS score 0–2) at 90 days after EVT for an average patient with acute ischemic stroke for each center was 36% in a center at the lower end (25th percentile) and 44% in a center at the higher end (75th percentile) of the outcome distribution (Figure 3). This decreased to 38% and 43%, respectively, after adjustment for modifiable center characteristics.

## Discussion

Our study shows that functional outcomes of acute ischemic stroke patients treated with EVT vary substantially between centers. The impact of center characteristics on between-center variation in outcomes was large. Adjustment for center characteristics decreased the relative difference in odds of a more favorable outcome between centers at the 25th and 75th percentile of the outcome distribution from 1.46 to 1.26 and decreased the absolute difference in estimated frequency for an average patient to achieve a good functional outcome from 8% to 5%. Even a small difference in outcome after EVT can have a major impact for an individual patient and also for society considering the incidence of stroke worldwide.

Other studies have shown that between-center variation in mortality and functional recovery rates after ischemic stroke can be explained to some extent by patient characteristics.^2–5^ However, the variation in outcome between regions and between centers remained largely unexplained. Furthermore, in these previous studies, the effect of center characteristics on between-center differences in outcome after stroke could not be established with certainty.^2,3,5^ Our study provides information on the association between center characteristics and functional outcome at a more detailed level in a unique set of acute stroke patients treated with EVT. In accordance with previous studies, the size and direction of the effect of center type and center size remained unclear.^2,3,5^ Possibly, other, not included patient characteristics, such as the Alberta Stroke Program Early Computed Tomography score or collateral score, may have differed between large and small EVT centers or between centers types. Also, the number of EVTs per interventionalist was not included in our analysis, let alone their competence in the intervention. Furthermore, we did not include the type of device used for EVT since this was not registered in the SITS-TBY. The influence of these factors on between-center differences in outcome after EVT need further study.

Our observation that between-center variation in time to reperfusion had a significant impact on the between-center variation in outcome is consistent with the observation from EVT trials that earlier treatment leads to a better functional outcome.^13^ In our study, time from groin puncture to reperfusion had a significant effect on between-center variation in outcomes, independent of the other center characteristics. Earlier studies have shown that greater experience and steeper learning curves of interventionalists can contribute to faster recanalization times and are likely to lead to improved functional outcomes.^14–16^ A meta-analysis showed that reducing time to treatment through interventions in the workflow of EVT, for example, by optimizing prehospital management, in-hospital transfer management, and teamwork, was associated with an increased likelihood of favorable outcome.^17^ A previous study has shown a modest favorable linear time trend over time towards a shorter door to first pass time in clinical practice.^18^ Future research should focus on the impact of interventions in the workflow of EVT, aimed at decreasing time to EVT, to improve outcome.

Our study has potential limitations. First, our results suggest that between-center variation in outcome after EVT for acute ischemic stroke could be partially explained by between-country differences. This between-country difference in outcome may be caused by our study design, since the 2 registries had a different method of data collection and had no blinding of outcome assessment. Furthermore, general differences between the 2 countries in organization of stroke health care before, during, and after hospital admission may have had an impact on the results. Second, our study cannot completely explain the observed between-center variation in outcomes in patients treated with EVT for acute ischemic stroke. Possibly other patient or center characteristics, not included in our study, also have an impact on between-center differences in outcomes. For example, low socioeconomic status is associated with a decreased survival and poor outcome in patients with ischemic stroke, independently of cardiovascular risk factors.^19–22^ Between-center differences in socioeconomic status of patients were not evaluated in our study. Although socioeconomic status could possibly explain between-center differences in outcome, this is a nonmodifiable patient characteristic. Adherence to stroke guidelines, however, is to some extent modifiable. Analysis of received, guideline-recommended, secondary stroke prevention treatments in 991 995 patients with stroke showed that almost one-quarter of patients received suboptimal care.^23^ This was also seen in a smaller study on quality of care parameters in acute stroke care, which showed that execution of recommended diagnostic investigations and medical treatment in the acute phase, subacute care, and performance of secondary prevention varied considerably between centers.^4^ To analyze the effect of guideline adherence, we could only include the percentage of patients treated >390 minutes after stroke onset, which was the recommended time window for EVT during the study period. Further research is needed to evaluate the influence of guideline adherence on between-center variation in outcome after EVT for acute ischemic stroke. A third limitation is the amount of loss to follow-up at 90 days resulting in missing mRS scores in 18% of patients. By performing multiple imputation using multiple variables including NIHSS at 24 to 48 hours, we tried to minimize bias in the estimates. Another variable with a relative large amount of missing data concerned the time of arrival at the primary stroke center. Therefore, missing arrival times were imputed, and the center characteristic concerning the time from first door to groin puncture was then computed.

Between-center variation in outcomes has not only been described in stroke, but also in other acute diseases, such as traumatic brain injury and myocardial infarction.^10,24–27^ A study of 9578 patients with moderate or severe traumatic brain injury showed that risk-adjusted odds of unfavorable outcome differed 3.3-fold between centers at the 2.5th and 97.5th percentile of the outcome distribution.^10^ A large observational study of 2 million patients with ST-segment–elevation myocardial infarction showed significant regional differences in in-hospital mortality, independent of patient characteristics and treatment selection.^24^ This study emphasized that our research focus should not only be directed on developing improved treatments but also on implementation of current evidence and guidelines in clinical practice.

The baseline characteristics of our included patients, with higher age and more frequent cardiovascular comorbidity than in the EVT trials, suggest that our study population reflects the acute ischemic stroke population eligible for EVT of many centers and countries. The large study population, treated in 33 interventions centers with varying center characteristics, makes the results of our study applicable to many centers and countries. This means that between-center variation in outcome after EVT for acute ischemic stroke needs to be acknowledged. We hope that addressing and visualizing differences between centers in outcome and in center characteristics may help to improve stroke care. This is already being applied in the European Stroke Organisation Enhancing and Accelerating Stroke Treatment program,^28^ and in the Dutch PERFEQTOS trial.^29^ To optimize the implementation of EVT, we need to determine the optimal combination of center characteristics, for example, balancing center size and time from onset to arrival at the intervention center. The optimal implementation differs between geographic countries and between regions, and thus is most easily addressed with modeling studies.^30^ However, we think that the association between the center characteristics we studied, such as time to treatment and volume, and outcome are generalizable to other settings. These association and their magnitudes should be taken into account when redesigning stroke systems.

We conclude that the proportion of patients with good functional outcome after EVT for acute ischemic stroke varies substantially between centers. This between-center variation in outcomes could be largely explained by center-specific characteristics, such as time to reperfusion. Improvement of these parameters may likely result in a decrease in center-specific differences, and an overall improvement in outcome of patients with acute ischemic stroke.

## Article Information

### Sources of Funding

The MR CLEAN (Multicenter Randomized Controlled Trial of Endovascular Treatment for Acute Ischemic Stroke in the Netherlands) registry was partly funded by TWIN Foundation, Erasmus MC University Medical Center, Maastricht University Medical Center, and Amsterdam UMC. Dr Mikulik was supported by the COST (European Cooperation in Science and Technology) Association, project no. CA18118, IRENE COST Action - Implementation Research Network in Stroke Care Quality; by the IRIS-TEPUS project no. LTC20051 from the INTER-EXCELLENCE INTER-COST program of the Ministry of Education, Youth, and Sports of the Czech Republic; and by STROCZECH within CZECRIN Large Research Infrastructure (no. LM2018128) funded by the state budget of the Czech Republic.

### Disclosures

Dr van der Worp served as a consultant to Bayer and LivaNova for which his institution received fees. Dr van der Worp received a research grant from Stryker via the CONTRAST consortium. Dr Majoie received funds from TWIN Foundation (related to this project, paid to institution); and from CVON/Dutch Heart Foundation, Stryker, European Commission (unrelated; all paid to institution). Dr Majoie is shareholder of Nico.lab, a company that focuses on the use of artificial intelligence for medical imaging analysis. Dr Dippel reports funding from the Dutch Heart Foundation, Brain Foundation Netherlands, The Netherlands Organisation for Health Research and Development, Health Holland Top Sector Life Sciences & Health, and unrestricted grants from Penumbra Inc, Stryker European Operations BV, Medtronic, Thrombolytic Science, LLC and Cerenovus for research, all paid to institution. The other authors report no conflicts.

### Supplemental Material

Table S1

## Supplementary Material



## References

[1] Goyal M Menon BK van Zwam WH Dippel DW Mitchell PJ Demchuk AM Dávalos A Majoie CB van der Lugt A de Miquel MA. Endovascular thrombectomy after large-vessel ischaemic stroke: a meta-analysis of individual patient data from five randomised trials. Lancet. 2016;387:1723–1731. 26898852 10.1016/S0140-6736(16)00163-X

[2] Bettger JP Thomas L Liang L Xian Y Bushnell CD Saver JL Fonarow GC Peterson ED. Hospital variation in functional recovery after stroke. Circ Cardiovasc Qual Outcomes. 2017;10:e002391. doi: 10.1161/CIRCOUTCOMES.115.002391 28096203 10.1161/CIRCOUTCOMES.115.002391

[3] Fonarow GC Smith EE Reeves MJ Pan W Olson D Hernandez AF Peterson ED Schwamm LH; Get With The Guidelines Steering Committee and Hospitals. Hospital-level variation in mortality and rehospitalization for medicare beneficiaries with acute ischemic stroke. Stroke. 2011;42:159–166. doi: 10.1161/STROKEAHA.110.601831 21164109 10.1161/STROKEAHA.110.601831

[4] Lingsma HF Dippel DW Hoeks SE Steyerberg EW Franke CL van Oostenbrugge RJ de Jong G Simoons ML Scholte Op Reimer WJ; Netherlands Stroke Survey investigators. Variation between hospitals in patient outcome after stroke is only partly explained by differences in quality of care: results from the Netherlands Stroke Survey. J Neurol Neurosurg Psychiatry. 2008;79:888–894. doi: 10.1136/jnnp.2007.137059 18208861 10.1136/jnnp.2007.137059

[5] Thompson MP Zhao X Bekelis K Gottlieb DJ Fonarow GC Schulte PJ Xian Y Lytle BL Schwamm LH Smith EE. Regional variation in 30-day ischemic stroke outcomes for medicare beneficiaries treated in get with the guidelines-stroke hospitals. Circ Cardiovasc Qual Outcomes. 2017;10:e003604. doi: 10.1161/CIRCOUTCOMES.117.003604 28798017 10.1161/CIRCOUTCOMES.117.003604

[6] Berkhemer OA Fransen PS Beumer D van den Berg LA Lingsma HF Yoo AJ Schonewille WJ Vos JA Nederkoorn PJ Wermer MJ; MR CLEAN Investigators. A randomized trial of intraarterial treatment for acute ischemic stroke. N Engl J Med. 2015;372:11–20. doi: 10.1056/NEJMoa1411587 25517348 10.1056/NEJMoa1411587

[7] Jansen IGH Mulder MJHL Goldhoorn RB; MR CLEAN Registry investigators. Endovascular treatment for acute ischaemic stroke in routine clinical practice: prospective, observational cohort study (MR CLEAN Registry). BMJ. 2018;360:k949. doi: 10.1136/bmj.k949 29523557 10.1136/bmj.k949 PMC5844245

[8] Volny O Krajina A Belaskova S Bar M Cimflova P Herzig R Sanak D Tomek A Köcher M Rocek M. Mechanical thrombectomy performs similarly in real world practice: a 2016 nationwide study from the Czech Republic. J Neurointerv Surg. 2018;10:741–745. doi: 10.1136/neurintsurg-2017-013534 29146830 10.1136/neurintsurg-2017-013534

[9] von Elm E Altman DG Egger M Pocock SJ Gøtzsche PC Vandenbroucke JP; STROBE Initiative. The Strengthening the Reporting of Observational Studies in Epidemiology (STROBE) statement: guidelines for reporting observational studies. Lancet. 2007;370:1453–1457. doi: 10.1016/S0140-6736(07)61602-X 18064739 10.1016/S0140-6736(07)61602-X

[10] Lingsma HF Roozenbeek B Li B Lu J Weir J Butcher I Marmarou A Murray GD Maas AI Steyerberg EW. Large between-center differences in outcome after moderate and severe traumatic brain injury in the international mission on prognosis and clinical trial design in traumatic brain injury (IMPACT) study. Neurosurgery. 2011;68:601–7. discussion 607. doi: 10.1227/NEU.0b013e318209333b 21311293 10.1227/NEU.0b013e318209333b

[11] Donders AR van der Heijden GJ Stijnen T Moons KG. Review: a gentle introduction to imputation of missing values. J Clin Epidemiol. 2006;59:1087–1091. doi: 10.1016/j.jclinepi.2006.01.014 16980149 10.1016/j.jclinepi.2006.01.014

[12] Moons KG Donders RA Stijnen T Harrell FE Jr. Using the outcome for imputation of missing predictor values was preferred. J Clin Epidemiol. 2006;59:1092–1101. doi: 10.1016/j.jclinepi.2006.01.009 16980150 10.1016/j.jclinepi.2006.01.009

[13] Saver JL Goyal M van der Lugt A Menon BK Majoie CB Dippel DW Campbell BC Nogueira RG Demchuk AM Tomasello A; HERMES Collaborators. Time to treatment with endovascular thrombectomy and outcomes from ischemic stroke: a meta-analysis. JAMA. 2016;316:1279–1288. doi: 10.1001/jama.2016.13647 27673305 10.1001/jama.2016.13647

[14] Benardete EA Nair AK. Endovascular stroke therapy results improve over time: the ‘learning curve’ at a new comprehensive stoke center. Cerebrovasc Dis Extra. 2014;4:235–242. doi: 10.1159/000370060 26225135 10.1159/000370060 PMC4347296

[15] Eesa M Burns PA Almekhlafi MA Menon BK Wong JH Mitha A Morrish W Demchuk AM Goyal M. Mechanical thrombectomy with the Solitaire stent: is there a learning curve in achieving rapid recanalization times? J Neurointerv Surg. 2014;6:649–651. doi: 10.1136/neurintsurg-2013-010906 24151114 10.1136/neurintsurg-2013-010906

[16] Nishi H Ishii A Nakahara I Matsumoto S Sadamasa N Kai Y Ishibashi R Yamamoto M Morita S Nagata I. Different learning curves between stent retrieval and a direct aspiration first-pass technique for acute ischemic stroke. J Neurosurg. 2018;129:1456–1463. doi: 10.3171/2017.6.JNS17872 29303452 10.3171/2017.6.JNS17872

[17] Janssen PM Venema E Dippel DWJ. Effect of workflow improvements in endovascular stroke treatment. Stroke. 2019;50:665–674. doi: 10.1161/STROKEAHA.118.021633 30776995 10.1161/STROKEAHA.118.021633

[18] Menon BK Xu H Cox M Saver JL Goyal M Peterson E Xian Y Matsuoka R Jehan R Yavagal D. Components and trends in door to treatment times for endovascular therapy in get with the guidelines-stroke hospitals. Circulation. 2019;139:169–179. doi: 10.1161/CIRCULATIONAHA.118.036701 30586703 10.1161/CIRCULATIONAHA.118.036701

[19] Kapral MK Fang J Chan C Alter DA Bronskill SE Hill MD Manuel DG Tu JV Anderson GM. Neighborhood income and stroke care and outcomes. Neurology. 2012;79:1200–1207. doi: 10.1212/WNL.0b013e31826aac9b 22895592 10.1212/WNL.0b013e31826aac9b PMC3440450

[20] Marshall IJ Wang Y Crichton S McKevitt C Rudd AG Wolfe CD. The effects of socioeconomic status on stroke risk and outcomes. Lancet Neurol. 2015;14:1206–1218. doi: 10.1016/S1474-4422(15)00200-8 26581971 10.1016/S1474-4422(15)00200-8

[21] Skyrud KD Vikum E Hansen TM Kristoffersen DT Helgeland J. Hospital variation in 30-day mortality for patients with stroke; the impact of individual and municipal socio-demographic status. J Am Heart Assoc. 2019;8:e010148. doi: 10.1161/JAHA.118.010148 31306031 10.1161/JAHA.118.010148 PMC6662128

[22] Vivanco-Hidalgo RM Ribera A Abilleira S. Association of socioeconomic status with ischemic stroke survival. Stroke. 2019;50:3400–3407. doi: 10.1161/STROKEAHA.119.026607 31610765 10.1161/STROKEAHA.119.026607

[23] Allen NB Kaltenbach L Goldstein LB Olson DM Smith EE Peterson ED Schwamm L Lichtman JH. Regional variation in recommended treatments for ischemic stroke and TIA: Get with the Guidelines–Stroke 2003-2010. Stroke. 2012;43:1858–1864. doi: 10.1161/STROKEAHA.112.652305 22588262 10.1161/STROKEAHA.112.652305

[24] Kolte D Khera S Aronow WS Mujib M Palaniswamy C Ahmed A Frishman WH Fonarow GC. Regional variation across the United States in management and outcomes of ST-elevation myocardial infarction: analysis of the 2003 to 2010 nationwide inpatient sample database. Clin Cardiol. 2014;37:204–212. doi: 10.1002/clc.22250 24477863 10.1002/clc.22250 PMC6649537

[25] Laskey W Spence N Zhao X Mayo R Taylor R Cannon CP Hernandez AF Peterson ED Fonarow GC. Regional differences in quality of care and outcomes for the treatment of acute coronary syndromes: an analysis from the get with the guidelines coronary artery disease program. Crit Pathw Cardiol. 2010;9:1–7. doi: 10.1097/HPC.0b013e3181cdb5a5 20215903 10.1097/HPC.0b013e3181cdb5a5

[26] Vallabhajosyula S Patlolla SH Dunlay SM Prasad A Bell MR Jaffe AS Gersh BJ Rihal CS Holmes DR Jr Barsness GW. Regional variation in the management and outcomes of acute myocardial infarction with cardiogenic shock in the United States. Circ Heart Fail. 2020;13:e006661. doi: 10.1161/CIRCHEARTFAILURE.119.006661 32059628 10.1161/CIRCHEARTFAILURE.119.006661 PMC7027926

[27] Greene NH Kernic MA Vavilala MS Rivara FP. Variation in adult traumatic brain injury outcomes in the United States. J Head Trauma Rehabil. 2018;33:E1–E8. doi: 10.1097/HTR.0000000000000306 10.1097/HTR.0000000000000306 PMC5647202 28422899

[28] Mikulík R Caso V Bornstein NM Svobodová V Pezzella FR Grecu A Simsic S Gdovinova Z Członkowska A Mishchenko TS. Enhancing and accelerating stroke treatment in Eastern European region: methods and achievement of the ESO EAST program. Eur Stroke J. 2020;5:204–212. doi: 10.1177/2396987319897156 32637654 10.1177/2396987319897156 PMC7313365

[29] Den Hartog SJ Amini M van Leeuwen N Kuhrij LS Nederkoorn PJ Lingsma HF van der Lugt AAD Dippel DWJ Roozenbeek B. Performance feedback on quality of care in hospitals performing thrombectomy for ischemic stroke (PERFEQTOS trial). European Stroke Journal. 2019;4:790–821.

[30] Venema E Burke JF Roozenbeek B Nelson J Lingsma HF Dippel DWJ Kent DM. Prehospital triage strategies for the transportation of suspected stroke patients in the United States. Stroke. 2020;51:3310–3319. doi: 10.1161/STROKEAHA.120.031144 33023425 10.1161/STROKEAHA.120.031144 PMC7587242

