# Supplementary material for: Between-Center Variation in Outcome After Endovascular Treatment of Acute Stroke: Analysis of Two Nationwide Registries
Source: Circ Cardiovasc Qual Outcomes. 2022 Jan 31;15(3):e008180. doi: 10.1161/CIRCOUTCOMES.121.008180 (PMC8920023; doi:10.1161/CIRCOUTCOMES.121.008180)
Supplement: Supplementary file 2 [file hcq-15-e008180-s002.pdf]

SUPPLEMENTAL MATERIAL

Between-Center Variation in Outcome after Endovascular Treatment of Acute Stroke: Analysis of Two Nationwide Registries

Paula M. Janssen, MD, Katrine van Overhagen, MD, Jan Vinklárek, MD, Bob Roozenbeek, MD, PhD, H. Bart van der Worp, MD, PhD, Charles B. Majoie, MD, PhD, Michal Bar, MD, PhD, David Černík, MD, Roman Herzig, MD, PhD, Lubomir Jurák, MD, PhD, Svatopluk Ostrý, MD, PhD, Robert Mikulik, MD, PhD, Hester F. Lingsma, PhD, Diederik W.J. Dippel, MD, PhD, on behalf of the MR CLEAN Registry investigators and the SITS TBY Registry investigators from the Czech Republic.

Affiliations

Erasmus MC University Medical Center Rotterdam, Department of Neurology, Rotterdam, The Netherlands (PJ, KvO, BR, DD). St Anne's University Hospital, International Clinical Research Center and Department of Neurology, Brno, Czech Republic (JV, RM). Faculty of Medicine at Masaryk University, Brno, Czech Republic (JV, RM). Erasmus MC University Medical Center Rotterdam, Department of Radiology and Nuclear Medicine, Rotterdam, The Netherlands (BR). University Medical Center Utrecht, Department of Neurology and Neurosurgery, Brain Center, UMC Utrecht, The Netherlands (HBvdW). Amsterdam University Medical Center, location AMC, Department of Radiology and Nuclear Medicine, Amsterdam, the Netherlands (CM). Department of Neurology, University Hospital Ostrava, Czech Republic (MB). Faculty of Medicine at University Ostrava, Czech Republic (MB). Masaryk Hospital Ústí nad Labem - KZ a.s., Comprehensive Stroke Center - Department of Neurology, Ústí nad Labem, Czech Republic (DC). Comprehensive Stroke Center, University Hospital Hradec Králové, Czech Republic (RH). Charles University Faculty of Medicine in Hradec Králové, Hradec Králové, Czech Republic (RH). Regional Hospital Liberec, Neurocenter, Liberec, Czech Republic (LJ). Comprehensive Stroke Center, Department of Neurology, Hospital České Budějovice, a.s., České Budějovice, Czech Republic (SO). Department of Neurosurgery and Neurooncology, First Faculty of Medicine, Charles University in Prague and Military University Hospital Prague (SO). Erasmus MC University Medical Center Rotterdam, Department of Public Health, Rotterdam, The Netherlands (HL).

Table I. Effect of combined center characteristics on between-center variation in outcome after EVT for acute ischemic stroke, with adjustment for year of intervention

Variables included in the multilevel ordinal logistic regression model	Relative difference in odds of a more favorable outcome between a relatively better performing center (75th percentile) and a relatively worse performing center (25th percentile) (95% CI)	Relative difference in odds of a more favorable outcome between a relatively better performing center(75th percentile) and a relatively worse performing center (25th percentile) (95% CI)<br>*Including adjustment for "year of intervention" for all steps of the model*
Patient characteristics	1.46 (1.31-1.70)	1.42 (1.28-1.65)
Patient characteristics + set 1 center characteristics	1.41 (1.27-1.64)	1.37 (1.24-1.59)
Patient characteristics + set 1 + set 2 center characteristics	1.30 (1.18-1.50)	1.30 (1.18-1.50)
Patient characteristics + set 1 and 2 center characteristics + country	1.26 (1.15-1.47)	1.24 (1.13-1.44)

Outcomes in the multilevel ordered logistic regression analysis were measured with the modified Rankin Scale (mRS) at 90 days. Center characteristics were added in two separate sets to the model based on relative modifiabilty.

* Patient characteristics were age, sex, previous ischemic stroke, diabetes mellitus, atrial fibrillation, hypercholesterolemia, hypertension, smoking, occlusion side, occlusion segment, baseline National Institutes of Health Stroke Scale score, and pre-stroke mRS score.

EVT = endovascular thrombectomy; CI = confidence interval.

Appendix

MR CLEAN Registry Investigators – group authors

Executive committee: Diederik W.J. Dippel[1];Aad van der Lugt[2];Charles B.L.M. Majoie[3];Yvo B.W.E.M. Roos[4];Robert J. van Oostenbrugge[5];Wim H. van Zwam[6];Jelis Boiten[14];Jan Albert Vos[8]

Study coordinators: Ivo G.H. Jansen[3];Maxim J.H.L. Mulder[1,2];Robert- Jan B. Goldhoorn[5,6];Kars C.J. Compagne[2];Manon Kappelhof[3];Josje Brouwer[4];Sanne J. den Hartog[1,2,40];Wouter H. Hinsenveld [5,6];

Local principal investigators: Diederik W.J. Dippel[1];Bob Roozenbeek[1];Aad van der Lugt[2];Adriaan C.G.M. van Es[2];Charles B.L.M. Majoie[3];Yvo B.W.E.M. Roos[4];Bart J. Emmer[3];Jonathan M. Coutinho[4];Wouter J. Schonewille[7];Jan Albert Vos[8]; Marieke J.H. Wermer[9];Marianne A.A. van Walderveen[10];Julie Staals[5];Robert J. van Oostenbrugge[5];Wim H. van Zwam[6];Jeannette Hofmeijer[11];Jasper M. Martens[12];Geert J. Lycklama à Nijeholt[13];Jelis Boiten[14];Sebastiaan F. de Bruijn[15];Lukas C. van Dijk[16];H. Bart van der Worp[17];Rob H. Lo[18];Ewoud J. van Dijk[19];Hieronymus D. Boogaarts[20];J. de Vries[22];Paul L.M. de Kort[21]; Julia van Tuijl[21] ; Jo P. Peluso[26];Puck Fransen[22];Jan S.P. van den Berg[22];Boudewijn A.A.M. van Hasselt[23];Leo A.M. Aerden[24];René J. Dallinga[25];Maarten Uyttenboogaart[28];Omid Eschgi[29];Reinoud P.H. Bokkers[29];Tobien H.C.M.L. Schreuder[30];Roel J.J. Heijboer[31];Koos Keizer[32];Lonneke S.F. Yo[33];Heleen M. den Hertog[22];Emiel J.C. Sturm[35]; Paul J.A.M. Brouwers[34]

Imaging assessment committee: Charles B.L.M. Majoie[3](chair);Wim H. van Zwam[6];Aad van der Lugt[2];Geert J. Lycklama à Nijeholt[13];Marianne A.A. van Walderveen[10];Marieke

E.S. Sprengers[3];Sjoerd F.M. Jenniskens[27];René van den Berg[3];Albert J. Yoo[38];Ludo F.M. Beenen[3];Alida A. Postma[6];Stefan D. Roosendaal[3];Bas F.W. van der Kallen[13];Ido R. van den Wijngaard[13];Adriaan C.G.M. van Es[2];Bart J. Emmer[,3];Jasper M. Martens[12]; Lonneke S.F. Yo[33];Jan Albert Vos[8]; Joost Bot[36], Pieter-Jan van Doormaal[2]; Anton Meijer[27];Elyas Ghariq[13]; Reinoud P.H. Bokkers[29];Marc P. van Proosdij[37];G. Menno Krietemeijer[33];Jo P. Peluso[26];Hieronymus D. Boogaarts[20];Rob Lo[18];Dick Gerrits[35];Wouter Dinkelaar[2]Auke P.A. Appelman[29];Bas Hammer[16];Sjoert Pegge[27];Anouk van der Hoorn[29];Saman Vinke[20].

Writing committee: Diederik W.J. Dippel[1](chair);Aad van der Lugt[2];Charles B.L.M. Majoie[3];Yvo B.W.E.M. Roos[4];Robert J. van Oostenbrugge[5];Wim H. van Zwam[6];Geert J. Lycklama à Nijeholt[13];Jelis Boiten[14];Jan Albert Vos[8];Wouter J. Schonewille[7];Jeannette Hofmeijer[11];Jasper M. Martens[12];H. Bart van der Worp[17];Rob H. Lo[18]

Adverse event committee: Robert J. van Oostenbrugge[5](chair);Jeannette Hofmeijer[11];H. Zwenneke Flach[23]

Trial methodologist: Hester F. Lingsma[40]

Research nurses / local trial coordinators: Naziha el Ghannouti[1];Martin Sterrenberg[1];Wilma Pellikaan[7];Rita Sprengers[4];Marjan Elfrink[11];Michelle Simons[11];Marjolein Vossers[12];Joke de Meris[14];Tamara Vermeulen[14];Annet Geerlings[19];Gina van Vemde[22];Tiny Simons[30];Gert Messchendorp[28];Nynke Nicolaij[28];Hester Bongenaar[32];Karin Bodde[24];Sandra Kleijn[34];Jasmijn Lodico[34]; Hanneke Droste[34];Maureen Wollaert[5];Sabrina Verheesen[5];D. Jeurrissen[5];Erna Bos[9];Yvonne

Drabbe[15];Michelle Sandiman[15];Nicoline Aaldering[11];Berber Zweedijk[17];Jocova Vervoort[21];Eva Ponjee[22];Sharon Romviel[19];Karin Kanselaar[19];Denn Barning[10].

PhD / Medical students: Esmee Venema[40]; Vicky Chalos[1,40]; Ralph R. Geuskens[3]; Tim van Straaten[19];Saliha Ergezen[1]; Roger R.M. Harmsma[1]; Daan Muijres[1]; Anouk de Jong[1];Olvert A. Berkhemer[1,3,6];Anna M.M. Boers[3,39]; J. Huguet[3];P.F.C. Groot[3];Marieke A. Mens[3];Katinka R. van Kranendonk[3];Kilian M. Treurniet[3];Manon L. Tolhuisen[3,39];Heitor Alves[3];Annick J. Weterings[3],Eleonora L.F. Kirkels[3],Eva J.H.F. Voogd[11];Lieve M. Schupp[3];Sabine L. Collette[28,29];Adrien E.D. Groot[4];Natalie E. LeCouffe[4];Praneeta R. Konduri[39];Haryadi Prasetya[39];Nerea Arrarte-Terreros[39];Lucas A. Ramos[39].

List of affiliations MR CLEAN Registry Investigators – group authors

Department of Neurology[1], Radiology[2], Public Health[40], Erasmus MC University Medical Center;

Department of Radiology and Nuclear Medicine[3], Neurology[4], Biomedical Engineering & Physics[39], Amsterdam UMC, University of Amsterdam, Amsterdam;

Department of Neurology[5], Radiology[6], Maastricht University Medical Center and Cardiovascular Research Institute Maastricht (CARIM);

Department of Neurology[7], Radiology[8], Sint Antonius Hospital, Nieuwegein;

Department of Neurology[9], Radiology[10], Leiden University Medical Center;

Department of Neurology[11], Radiology[12], Rijnstate Hospital, Arnhem;

Department of Radiology[13], Neurology[14], Haaglanden MC, the Hague;

Department of Neurology[15], Radiology[16], HAGA Hospital, the Hague;

Department of Neurology[17], Radiology[18], University Medical Center Utrecht;

Department of Neurology[19], Neurosurgery[20], Radiology[27], Radboud University Medical Center, Nijmegen;

Department of Neurology[21], Radiology[26], Elisabeth-TweeSteden ziekenhuis, Tilburg;

Department of Neurology[22], Radiology[23], Isala Klinieken, Zwolle;

Department of Neurology[24], Radiology[25], Reinier de Graaf Gasthuis, Delft;

Department of Neurology[28], Radiology[29], University Medical Center Groningen;

Department of Neurology[30], Radiology[31], Atrium Medical Center, Heerlen;

Department of Neurology[32], Radiology[33], Catharina Hospital, Eindhoven;

Department of Neurology[34], Radiology[35], Medical Spectrum Twente, Enschede;

Department of Radiology[36], Amsterdam UMC, Vrije Universiteit van Amsterdam, Amsterdam;

Department of Radiology[37], Noordwest Ziekenhuisgroep, Alkmaar;

Department of Radiology[38], Texas Stroke Institute, Texas, United States of America.
